# Adjusting for reverse causation to estimate the effect of obesity on mortality after incident heart failure in the Atherosclerosis Risk in Communities (ARIC) study

**DOI:** 10.4178/epih.e2016025

**Published:** 2016-06-04

**Authors:** Maryam Shakiba, Hamid Soori, Mohammad Ali Mansournia, Seyed Saeed Hashemi Nazari, Yahya Salimi

**Affiliations:** 1 Department of Epidemiology, School of Public Health, Shahid Beheshti University of Medical Sciences, Tehran, Iran; 2 Interventional Cardiovascular Research Center, Guilan University of Medical Sciences, Rasht, Iran; 3 Safety Promotion and Injury Prevention Research Center, Shahid Beheshti University of Medical Sciences, Tehran, Iran; 4 Department of Epidemiology and Biostatistics, School of Public Health, Tehran University of Medical Sciences, Tehran, Iran; 5 Department of Epidemiology and Biostatistics, School of Public Health, Kermanshah University of Medical Sciences, Kermanshah, Iran

**Keywords:** Obesity, Heart failure, Mortality, Weight loss, Body mass index

## Abstract

**OBJECTIVES:**

The lower mortality rate of obese patients with heart failure (HF) has been partly attributed to reverse causation bias due to weight loss caused by disease. Using data about weight both before and after HF, this study aimed to adjust for reverse causation and examine the association of obesity both before and after HF with mortality.

**METHODS:**

Using the Atherosclerosis Risk in Communities (ARIC) study, 308 patients with data available from before and after the incidence of HF were included. Pre-morbid and post-morbid obesity were defined based on body mass index measurements at least three months before and after incident HF. The associations of pre-morbid and post-morbid obesity and weight change with survival after HF were evaluated using a Cox proportional hazard model.

**RESULTS:**

Pre-morbid obesity was associated with higher mortality (hazard ratio [HR], 1.61; 95% confidence interval [CI], 1.04 to 2.49) but post-morbid obesity was associated with increased survival (HR, 0.57; 95% CI, 0.37 to 0.88). Adjusting for weight change due to disease as a confounder of the obesity-mortality relationship resulted in the absence of any significant associations between post-morbid obesity and mortality.

**CONCLUSIONS:**

This study demonstrated that controlling for reverse causality by adjusting for the confounder of weight change may remove or reverse the protective effect of obesity on mortality among patients with incident HF.

## INTRODUCTION

Despite the fact that obesity is positively related to all-cause mortality in the general population [1,2], the “obesity paradox,” defined as a lower mortality rate of obese and overweight individuals compared to normal-weight individuals, has been recognized for various chronic diseases [3-5], including chronic heart failure (HF) [6,7]. Among some plausible explanations that have been presented, some authors have attributed this paradox to a higher metabolic reserve putatively present among obese patients [8,9]. In contrast, other authors have explained the obesity paradox as a result of bias due to reverse causation [10-12]. Briefly, this explanation proposes that low weight is a consequence of pre-existing illness resulting in weight loss, which can distort the true relationship between body weight and the risk of death [13-16]. Some strategies for avoiding this form of bias have been implemented in previous studies, such as performing research in occupational cohorts, restricting samples to healthy individuals who are less likely to have chronic diseases, following up for a long period of time, excluding early deaths that may have occurred due to pre-existing illness, and/or using maximum lifetime body mass index (BMI) [1,13,17,18]. All of these approaches have been implemented in the general population, but due to the limited size of available patients, these methods may have limited applicability for evaluating the obesity-mortality relationship in patients with chronic diseases. In this regard, pre-existing illness is also a confounding variable since it affects both body weight (through weight loss) and future mortality [15,19,20]. This study proposed that weight change due to HF was a variable that could serve as a confounder for the obesity-mortality relationship and that adjusting for it may eliminate the reverse causation bias due to pre-existing disease.

In light of the discordant opinions regarding the obesity-mortality relationship, we conducted an analysis using the Atherosclerosis Risk in Communities (ARIC) study to investigate the association between obesity and future mortality in patients with incident HF. One aim of this study was to examine the effect of reverse causation by controlling for weight change as confounder. The hypothesis was that if weight loss distorts the true association between body weight and mortality, the obesity-mortality relationship will disappear as a result of adjusting for weight change. The other aim of this study was to estimate the association of obesity both before and after the development of HF with mortality. If greater metabolic reserve contributes to lower mortality, then obesity before the development of HF would be associated with better survival.

## MATERIALS AND METHODS

### Study population

The ARIC study was a community-based prospective cohort study designed to investigate the causes of atherosclerosis. The study recruited 15,792 adults aged 45 years to 64 years at baseline who resided in four US communities. The first examinations of participants (visit 1) took place during 1987 to 1989, followed by three visits at 3-year intervals. The follow-up rates for the first, second, and third intervals were 91%, 82%, and 74%, respectively. At each visit, participants responded to an interviewer-administered questionnaire and underwent an extensive physical examination [21]. The current study was restricted to patients with incident HF through December 31, 2009. Among 2,169 patients with incident HF, 1,861 cases were excluded because HF developed after the fourth examination, meaning that no data were available after the development of disease. Therefore, the present study included 308 patients with data available both before and after the incidence of HF. The study sample had a 0% prevalence of HF at baseline. This study was approved by the institutional review board of the Shahid Beheshti University of Medical Sciences.

### Determination of heart failure and mortality outcomes

Incident HF was defined as death or hospitalization with an International Classification of Diseases, 9th revision (ICD-9) code for HF (i.e., any variation of 410) given the absence of prevalent HF at visit 1. HF cases were determined through annual telephone interviews regarding their experiences of hospitalization, reviewing hospital discharge lists, and a survey of the national death index and death certificate files. The latter two methods were performed through community-wide surveillance of the communities involved in the ARIC study. The response rate for telephone interviews was 93% to 96%. Mortality outcomes were evaluated in the same way.

### Covariates

For this study, demographic variables including sex, race (black or white), and education level (basic or no formal education, intermediate, and advanced education) were determined at visit 1. The data before and after the development of HF were considered to define pre-morbid and post-morbid variables. Premorbid variables were based the measurement of covariates ≥3 months before incident HF and post-morbid variables were defined as measurements of covariates ≥3 months after incident HF. On this basis, pre-morbid and post-morbid values for comorbidities including a history of cancer, coronary heart disease, stroke and chronic lung disease, as well as covariates including smoking and drinking status, diabetes mellitus, hypertension, and total serum cholesterol were obtained. Similarly, pre-morbid and post-morbid obesity were defined using BMI measurements from study visits occurring ≥3 months prior and ≥3 months after the date of incident HF, respectively. Due to the small sample of available patients, we categorized BMI into non-obese (BMI <30 kg/m^2^) and obese (BMI ≥30 kg/m^2^). Weight change was defined as percentage change in BMI and calculated as the change in BMI value divided by the original (pre-HF) values of BMI multiplied by 100. Weight change was then categorized based on a priori cutpoint [10]. A weight change of ≤-6% was classified as weight loss, a change of -6% to 6% was classified as stable weight, and a change of ≥6% was classified as weight gain. Smoking status was defined as positive if the subjects ever smoked or smoked cigarettes at the time of the study. Drinking status was considered as positive if subjects regularly drank at the time of the study or had ever consumed alcoholic beverages. Hypertension was defined as a systolic blood pressure ≥90 mmHg, a diastolic blood pressure ≥140 mmHg, or the use of medication for high blood pressure in the previous two weeks. Diabetes mellitus was defined as a non-fasting blood glucose ≥200 mg/dL, a fasting blood glucose level ≥126 mg/dL, or the use of diabetes medication in the previous two weeks. Fasting blood glucose and total serum cholesterol were measured after overnight fasting. Cholesterol level was categorized using the common cutpoint of ≥240 mg/dL.

### Statistical analysis

Baseline data were described as mean (standard deviation, SD) or frequency (percentage) and compared using the t-test or the chi-square test. The pre-morbid and post-morbid measurements of risk factors were compared using the paired t-test or the McNemar test. Correlations between pre-morbid and post-morbid covariates were evaluated using the Spearman test. In the multivariate adjusted model, pre-morbid covariates were dropped if they were highly correlated with post-morbid covariate values (r≥0.80). The endpoint of interest was time to all-cause mortality after incident HF. A Cox proportional hazard model with age as the underlying time scale was used to examine the effect of pre-morbid and post-morbid obesity and other covariates on survival. The proportionality assumption was assessed using a graphical approach and testing the slope of Schoenfeld residuals. The Cox model used the date of birth as the origin of the time scale. In order to calculate weight change as a confounder, we used each participant’s weight before the potential onset of disease as their original weight. Weight change naturally occurs before post-morbid obesity, so it was not an intermediate variable between the exposure of interest and outcome. All statistical analyses were performed using Stata version 13.0 (StataCorp, College Station, TX, USA).

## RESULTS

The median and interquartile range of the duration from the previous visit to incident HF was 24.5 (14 to 31) months and from incident HF to the post-visit follow-up was 17 (9 to 27) months. Among 308 patients with incident HF and data available for the pre-morbid and post-morbid covariates, 47% were obese pre-morbidity, 61% were male, 33% were black, and 44% had a basic education. The mean age at the incidence of HF was 61 years old (SD, 5.9 years). A total of 3,027 person-years were incorporated into this study, during which 231 deaths occurred. The median follow-up was 9.8 years and the death rate was 76 per 1,000 person-years. Table 1 presents the pre-morbid and post-morbid characteristics of patients with incident HF according to pre-morbid and post-morbid obesity. Pre-morbid obese patients were younger, more likely to be black, and had a higher percentage of diabetes and hypertension. In contrast, the percentage of smoking status was lower in obese patients than in non-obese patients. No significant differences were found in education levels, drinking status, high cholesterol value, history of coronary heart disease, cancer, stroke, chronic lung disease, and weight change categories. Post-morbidity, the overall prevalence of smoking and drinking decreased. Post-morbid obese patients also had a lower prevalence of drinking and smoking than non-obese patients. However, the prevalence of diabetes and hypertension increased, and these conditions were more common in post-morbid obese patients than in non-obese patients. The mean percentage of weight change was -2.71% (minimum, -34%; maximum, 90%). The percentages of patients who experienced weight loss and weight gain were 30% and 20%, respectively. A significant difference was found in the death rate among the three weight change categories (p=0.02), with higher crude death rates in patients who experienced weight loss (95 per 1,000 person-years), compared to those with a stable weight and those who underwent weight gain (70 and 67 per 1,000 person-years, respectively).

**Figure 1.**
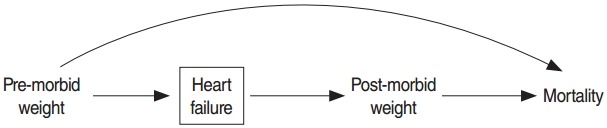
Directed acyclic graph representing the relationships of pre-morbid and post-morbid weight with mortality. The square box indicates that the study focused on heart failure.

A multivariate adjusted model was used to determine the associations of pre-morbid and post-morbid obesity with mortality. Based on subject matter knowledge and the graphical representation of variables as a directed acyclic graph in Figure 1, weight before the development of HF may have confounded the relation between post-morbid weight and mortality. Weight after the development of HF was also a strong mediator for the effect of pre-morbid obesity on mortality. Therefore, in order to estimate the direct effect of pre-morbid obesity, we adjusted for post-morbid obesity and other covariates. The results of the multivariate adjusted model are presented in Table 2. It was found that the hazard ratio (HR) of mortality in pre-morbid obese patients was significantly higher than in non-obese patients (HR, 1.61; 95% confidence interval [CI], 1.04 to 2.49), while post-morbid obesity was associated with a reduced risk of mortality (HR, 0.57; 95%CI, 0.37 to 0.88). In this model, post-morbid diabetes also significantly increased the risk of mortality. Pre-morbid diabetes was dropped from the model due to its high correlation (r=0.85) with post-morbid diabetes.

**Figure 2.**
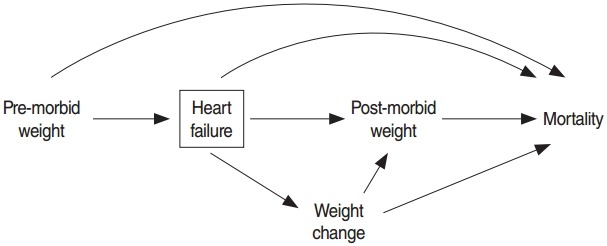
Directed acyclic graph representing weight change due to disease as a confounder for the relationship between post-morbid weight and mortality

According to the reverse causation hypothesis, as presented in Figure 2, weight change due to disease may confound the association of post-morbid obesity and mortality. Table 3 shows the distribution of weight change by post-morbid BMI category. A strong association was found between weight change and post-morbid obesity status, such that 40% of non-obese patients experienced weight loss, in contrast to only 18% of obese patients. The results of the univariate Cox model showed that a strong inverse association was present between weight change and mortality. The risk of death decreased by 3% with each 1-unit increment of the percentage change in BMI (HR, 0.97; 95% CI, 0.96 to 0.98). Table 4 shows the effect of post-morbid obesity on all-cause mortality adjusting for weight change and other pre-morbid and post-morbid covariates. In this model, post-morbid obesity was no longer a significant protective factor. The adjusted HR for weight change showed that patients who experienced weight loss had a 42% higher all-cause mortality (95% CI, 1.02 to 1.97), whereas patients who experienced weight gain had a 33% reduction in all-cause mortality compared to stable-weight patients (Table 4).

## DISCUSSION

In this study, reverse causation was revisited and evaluated using a new approach that treated weight change due to disease as a confounder. Our results demonstrate that reverse causation may result in the protective effect of post-morbid obesity on all-cause mortality in patients with incident HF. This conclusion is supported by the strong association we found between weight change due to disease and post-morbid obesity and the association of weight loss with all-cause mortality. Therefore, mortality in obese patients may be reduced due to their lower likelihood of having experienced weight loss. We used a relative measure for weight change in which all patients who lost more than 6% of their original BMI were categorized as experiencing weight loss, irrespective of their original weight.

The issue of reverse causation as a form of bias occurring in studies of obesity and mortality has generally been accounted for in the general population [1,13,17,18]. To our knowledge, though, this is the first study to adjust for reverse causation in patients with HF. In this study, we hypothesized that weight change due to HF could serve as a confounder for the obesity-mortality relationship. The main strength of this study was that weight change was calculated from the weight before any potential onset of disease. Hence, any changes in weight could attributed to the disease condition. Furthermore, since some cases of weight loss may have been caused by a reduction in edema that could have been present in the early stages of HF, calculating weight changes based on the weight before the development of disease would better reflect weight changes as a result of disease. The results of the current study showed an increased risk of all-cause mortality for patients who experienced weight loss compared to stable-weight patients, independently of post-morbid obesity and other covariates. This finding is consistent with those of previous studies that have found strong independent effects of weight loss during the course of disease on mortality [8,10-12,22-25]. Our results support the importance of reverse causation as a plausible explanation for the lower mortality rates of obese patients. After adjustment for weight changes due to disease, the significant protective association of obesity on mortality disappeared.

In this study, pre-morbid obese patients who eventually developed HF had a higher risk of mortality than pre-morbid non-obese patients. This finding is in contrast with a previous report that found better survival rates among pre-morbid obese patients [26]. The main shortcoming of the previous study is that it did not consider the effect of the post-morbid weight as an indirect effect of pre-morbid obesity on mortality. The development of edema as a result of salt and water retention in the early stages of HF and wasting due to cardiac cachexia in the later stage influence weight after the incidence of HF. Therefore, in order to estimate the direct effect of pre-morbid weight, weight after disease should be adjusted for. Our finding regarding the higher mortality rate of pre-morbid obese patients does not support the putative protective effect of higher metabolic reserve in obese patients. However, our result demonstrated support for the obesity paradox by documenting better survival associated with post-morbid obesity, disregarding the effect of weight change as a confounder.

Although the term “obesity paradox” is accepted by many scientific communities and listed in some guidelines for cardiovascular disease, it seems that some methodological flaws still persist in estimating the effect of obesity among patients suffering from chronic diseases [27]. This study highlighted the effect of weight change as an important confounder for the obesity-mortality relationship. In fact, the category of non-obese patients after disease development may include many patients who have experienced weight loss and have a higher risk of mortality, which erroneously makes obesity appear to be a protective factor [9,15].

This study suffers from some limitations. The identification of incident HF cases was only based on ICD-9 codes for hospitalizations and deaths due to HF, which were not validated, while outpatients with HF were included through responses from physicians using a form. However, the validation of ICD-9 codes by a subsequent medical record review following the ARIC criteria in the ARIC community surveillance study showed a sensitivity of 95% and a positive predictive value of 85%, which are both higher than the Framingham criteria [28]. Additionally, due to the small number of participants with data available before and after incident HF, pre-morbid and post-morbid variables were defined as events occurring at an interval of at least three months. Furthermore, BMI was categorized into two categories, comparing obese and non-obese patients. Therefore, we could not assess differences among all BMI categories, including overweight and underweight. Finally, this study has the inherent limitation of residual confounding due to unmeasured covariates, such as nutritional status and physical fitness, which could make associations a poor estimate of causal effects.

In this study, obese patients who developed HF had a higher risk of mortality than non-obese patients. Thus, greater metabolic reserve did not serve as a protective factor against mortality among obese patients. Post-morbid obesity was not a significant prognostic factor for mortality when the relationship was adjusted for weight change due to HF. Reverse causation may be a plausible explanation for the lower mortality risk of post-morbid obese patients.

## Figures and Tables

**Table 1.** Pre-morbid and post-morbid characteristics of 308 patients with available data before and after incident heart failure

	Pre-morbid obesity		p-value^1^	Post-morbid obesity		p-value	p-value^2^ (within group)
	No (n = 164)	Yes (n = 144)		No (n = 178)	Yes (n = 130)		
Age at baseline (yr)	62 (0.4)	60 (0.5)	0.001				
Sex (male)	110 (67)	77 (53)	0.01	111 (62)	76 (58)	0.45	
Race (black )	44 (26)	59 (41)	0.005	58 (32)	45 (34)	0.71	
Education level			0.14			0.83	
Basic	58 (35)	66 (46)		70 (39)	54 (41)		
Intermediate	64 (39)	51 (35)		66 (37)	49 (38)		
Advanced	42 (26)	27 (19)		42 (24)	27 (21)		
Smoking status	64 (39)	35 (25)	0.007	42 (24)	13 (11)	0.008	0.001
Drinking status	78 (48)	54 (38)	0.09	66 (38)	26 (20)	0.009	0.001
Diabetes mellitus	45 (27)	64 (46)	0.001	55 (32)	66 (58)	0.001	0.001
Hypertension	88 (54)	98 (71)	0.003	110 (64)	87 (76)	0.03	0.003
Total cholesterol (≥240 mg/dL)	41 (25)	32 (23)	0.74	28 (16)	19 (17)	0.83	0.002
History of coronary heart disease	49 (31)	39 (28)	0.58	106 (61)	57 (51)	0.11	
History of cancer	9 (5)	8 (6)	0.94	6 (3)	2 (2)	0.40	
History of stroke	10 (6)	8 (6)	0.85	18 (10)	13 (11)	0.73	
History of chronic lung disease	11 (7)	7 (5)	0.50	15 (9)	15 (13)	0.20	
Weight change			0.67				
Weight loss	47 (29)	47 (32)					
Stable weight	81 (49)	70 (49)					
Weight gain	36 (22)	27 (19)					

Values are presented as mean (standard deviation) or frequency (%). The risk factors and comorbidities were separately defined as pre-morbid and post-morbid covariates for pre-morbid and post-morbid obesity, respectively.

1 Chi-square or t-test.

2 McNemar or paired t-test.

**Table 2.** Crude and multivariate adjusted model for all-cause mortality in patients with incident heart failure

Covariates	No. of events (person-years)	Crude	p-value	Adjusted^1^	p-value
Sex			0.85		0.15
Female	88 (1,193)	1.00 (reference)		1.00 (reference)	
Male	143 (1,830)	0.97 (0.74, 1.27)		1.28 (0.91, 1.79)	
Race			0.001		0.16
Black	88 (872)	1.00 (reference)		1.00 (reference)	
White	143 (2,155)	0.59 (0.45, 0.78)		0.76 (0.52, 1.11)	
Pre-morbid obesity	107 (1,408)	1.12 (0.86, 1.45)	0.41	1.61 (1.04, 2.49)	0.03
Post-morbid obesity	88 (1,269)	0.89 (0.68, 1.16)	0.39	0.57 (0.37, 0.88)	0.01
Pre-morbid smoking	83 (966)	1.36 (1.03, 1.79)	0.03	1.13 (0.75, 1.70)	0.56
Post-morbid smoking	50 (509)	1.79 (1.29, 2.49)	0.001	1.59 (0.99, 2.57)	0.05
Pre-morbid drinking	92 (1,339)	0.83 (0.64, 1.09)	0.18	1.02 (0.70, 1.49)	0.90
Post-morbid drinking	65 (987)	0.81 (0.60, 1.09)	0.17	0.89 (0.59, 1.35)	0.60
Post-morbid diabetes	103 (1,064)	1.80 (1.37, 2.37)	0.001	1.78 (1.28, 2.47)	0.001
Pre-morbid hypertension	146 (1,665)	1.43 (1.09, 1.88)	0.01	1.12 (0.78, 1.62)	0.53
Post-morbid hypertension	152 (1,881)	1.34 (0.99, 1.81)	0.06	0.94 (0.62, 1.41)	0.75
Pre-morbid (cholesterol ≥240 mg/dL)	59 (652)	1.44 (1.06, 1.94)	0.02	1.32 (0.91, 1.98)	0.14
Post-morbid (cholesterol ≥240 mg/dL)	40 (439)	1.31 (0.93, 1.85)	0.12	0.85 (0.54, 1.34)	0.49

Values are presented as hazard ratio (95% confidence interval).

1 Age was fine-adjusted as the time scale.

**Table 3.** Associations between post-morbid obesity status and weight change among patients with incident heart failure

	Non-obese (n = 178)	Obese (n = 130)	p-value
Weight loss	71 (40)	23 (18)	0.001
Stable weight	85 (48)	66 (51)	
Weight gain	22 (12)	41(31)	

Values are presented as frequency (%).

**Table 4.** Crude and multivariate adjusted model for the effect of post-morbid obesity on all-cause mortality, adjusting for weight change and other pre-morbid and post-morbid covariates

Covariates	No. of events (person-years)	Crude	p-value	Adjusted^1^	p-value
Weight change					
Stable weight	108 (1,549)	1.00 (reference)		1.00 (reference)	
Weight loss	80 (833)	1.43 (1.07, 1.91)	0.01	1.42 (1.02, 1.97)	0.03
Weight gain	43 (644)	0.93 (0.65, 1.33)	0.69	0.67 (0.41, 1.09)	0.11
Post-morbid obesity	88 (1,269)	0.89 (0.69, 1.17)	0.69	0.80 (0.57, 1.12)	0.19

HR, hazard ratio; CI, confidence interval.

1 Hazard ratios were adjusted for pre-morbid and post-morbid smoking and drinking status, hypertension, total cholesterol, sex, race, and post-morbid diabetes.
